# Electrocardiographic Associations Seen with Obstructive Sleep Apnea

**DOI:** 10.1155/2019/9704785

**Published:** 2019-02-27

**Authors:** Shyam Shankar, Sushilkumar Satish Gupta, Geurys Rojas-Marte, Selma Demir, Abhinav Saxena, Chukwudi Obiagwu, Nidhi Aggarwal, Anand Kumar Rai, Stephan Kamholz, Vijay Shetty, Yizhak Kupfer

**Affiliations:** ^1^Fellow, Department of Pulmonary and Critical Care Medicine, Maimonides Medical Center, Brooklyn, New York, USA; ^2^Fellow, Department of Cardiology, Rutgers, New Jersey Medical School, Newark, New Jersey, USA; ^3^Attending, Department of Medicine, Baystate Medical Center, Springfield, Massachusetts, USA; ^4^Fellow, Department of Cardiology, Maimonides Medical Center, Brooklyn, New York, USA; ^5^Attending, Department of Pulmonary and Critical Care, Maimonides Medical Center, Brooklyn, New York, USA; ^6^Chairman, Department of Internal Medicine, Maimonides Medical Center, Brooklyn, New York, USA; ^7^Attending, Department of Cardiology, Maimonides Medical Center, Brooklyn, New York, USA; ^8^Director, Department of Critical Care Medicine, Maimonides Medical Center, Brooklyn, New York, USA

## Abstract

**Background:**

Obstructive sleep apnea (OSA) is a chronic respiratory disorder associated with repeated nocturnal partial or complete collapse that is often underdiagnosed and associated with multiple comorbidities. The association between specific features on an electrocardiogram and OSA has not been well studied. This retrospective study attempts to bridge this gap in knowledge.

**Methods:**

A total of 265 patients' medical records were reviewed retrospectively. Specific features of their electrocardiograms and their association with the severity of OSA were studied from April 2014 to May 2016. 215 patients were included in the final analysis. Tests of group difference between OSA patients and controls were done using student's t-tests for continuous variables and using chi-square tests for categorical outcomes. Multivariate tests of differences between OSA and control patients were done using logistic regression to control for possible confounding factors.

**Results:**

A total of 215 patients with diagnosed OSA and 41 controls in whom OSA was ruled out using polysomnography were compared. Males were more likely to present with OSA than females (93 % versus 76 %; p < 0.001). OSA patients were also significantly older: 52.18 ± 14.04 versus 44.55 ± 14.64; p = 0.002. Deep S waves in V5-6 (p=0.014) and RS pattern with Deep S waves in leads I and AVF (p=0.017) were both significantly associated with OSA based on univariate comparisons. These findings lost significance in the multivariate analysis.

**Conclusion:**

The idea of using an electrocardiogram in aiding in the assessment of OSA is attractive and feasible, as it is a safe, noninvasive, and cost-effective method. Our results can be used for early risk stratification in patients with OSA.

## 1. Introduction

Obstructive sleep apnea (OSA) is a common disorder characterized by repetitive episodes of nocturnal breathing cessation due to upper airway collapse. The incidence of OSA from population-based studies has ranged from 4.9% to 15% [1–3]. OSA can be diagnosed through polysomnography. During polysomnography oronasal airflows were measured. We used oronasal thermal sensor to identify apneas and nasal pressure transducer to identify hypopneas. The Wisconsin Sleep Cohort Study determined the prevalence of OSA to be 9% in females and 24% in men in a random sample of 602 middle aged state employees in Wisconsin [4].

STOP BANG questionnaires and the Berlin questionnaires have been used in screening for patients with obstructive sleep apnea. Despite the estimated prevalence, the actual number of patients who remain undiagnosed is potentially high. The association of OSA with several cardiovascular diseases [4], including hypertension [5], congestive heart failure [6], and atrial fibrillation [7] has made OSA a multifaceted disease requiring a multidisciplinary approach to management.

Several studies have been conducted to determine specific electrocardiographic associations with OSA, with often differing findings, looking particularly at QT intervals, QRS duration, P wave duration, and T wave alternans patterns [8–11].

The aim of this study was to determine the relationship between specific electrocardiographic features and OSA. This may aid in early risk stratification and primary prevention of cardiovascular diseases in general population based on electrocardiographic features on a 12 lead ECG, a simple, cost-effective, and noninvasive test.

## 2. Methods

A retrospective case-control study was performed involving patients who underwent a monitored sleep study and were enrolled from April 2014 to May 2016. Patients with underlying structural heart disease, cor pulmonale, and arrhythmias were excluded. Appropriate Institutional Review Board (IRB) approval was obtained from the Maimonides Medical Center IRB committee.

Demographic data, cardiovascular risk factors, and various electrocardiographic features from a standard 12 lead electrocardiogram (ECG) were recorded. These electrocardiographic features were specifically atrial fibrillation (Atrial fibrillation), left atrial enlargement (LAE), right ventricular hypertrophy (RVH), left ventricular hypertrophy (LVH), deep S wave in V5/6, left anterior hemiblock, premature atrial contractions (PAC), premature ventricular contractions (PVC), atrioventricular blocks, right bundle branch block (RBBB), QTc duration, and PR segment duration. Standard definitions of LAE, LVH, RVH, RBBB, and deep S wave were used to identify ECG abnormalities (Supplement A).

## 3. Statistical Analysis

Continuous variables (e.g., age) were described in terms of mean ± standard deviation while categorical variables (e.g., gender) were characterized in terms of frequency (percent). Test of group difference between OSA patients and controls were done using student's t-tests for continuous variables and using chi square tests for categorical outcomes. Multivariate tests of differences between OSA and control patients were done using logistic regression to control for possible confounding factors. All statistical tests assumed a significance level < 0.05. All analyses were done using SPSS 23.0 (IBM Corp., Armonk, NY).

## 4. Results

A total of 215 patients with diagnosed OSA and 41 controls in whom OSA was ruled out by using polysomnography were compared. Males were more likely to present with OSA than females (93 % versus 76 %; p < 0.001). OSA patients were also significantly older: 52.18 ± 14.04 versus 44.55 ± 14.64; p = 0.002 (Table 1). There were no other statistically significant demographic risk factor differences between the OSA cases and the control groups.

Table 2 highlights the ECG clinical parameters found in both groups, cases, and controls. Deep S waves in V5-6 (p=0.014) and RS pattern with Deep S waves in leads I and AVF (p=0.017) were both found to be statistically significant with OSA based on univariate comparisons. Other parameters including atrial fibrillation (p=0.413) and left atrial enlargement (p=0.157) were not found to be statistically significant with OSA.

## 5. Discussion

Obstructive sleep apnea is characterized by repetitive periods of pharyngeal airway collapse leading to periods of apnea or hypopnea followed by arousal from sleep leading to termination of the apneic event. OSA has been shown to be independently associated with the pathogenesis of several cardiovascular diseases, hypertension, congestive heart failure, arrhythmias, and stroke [4–8]. Hypoxemia and hypercapnia from these apneic episodes, along with imbalance between the sympathetic and the parasympathetic regulation, have been shown to increase nocturnal blood pressure readings and arrhythmias. Gami et al. found that individuals with OSA have an increased incidence of sudden cardiac death as compared to those without OSA [12].

Approximately 50% patients with OSA are diagnosed with hypertension and 30% hypertensive patients have OSA [5]. Nocturnal arrhythmias occur in approximately 50% of OSA patients, and the common ones are PAC's and PVC's, while the less common are atrial fibrillation and nonsustained ventricular tachycardia [7, 8, 11]. In patients less than 65 years, OSA independently correlates with the incidence of atrial fibrillation within 5 years of its diagnosis [7]. Hypoxemia, imbalance between the sympathetic and parasympathetic activation, transmural pressure changes, and systemic inflammation have all been proposed as possible mechanisms for the pathogenesis of atrial fibrillation in these patients [7].

Several studies highlight the association of OSA with atrial fibrillation. Guilleminault et al. demonstrated 3% prevalence of atrial fibrillation in moderate to severe obstructive sleep apnea [13]. Another study showed a higher prevalence of 4.8% of atrial fibrillation in individuals with sleep breathing disorders [14]. However, our study demonstrates no significant association of atrial fibrillation and left atrial enlargement with OSA. It is possible that our population subset was diagnosed with OSA early. Another reason for the lack of association between atrial fibrillation and OSA in our study might be the fact that these patients may have paroxysmal atrial fibrillation, which comprises most cases of atrial fibrillation that may have been missed on a routine ECG.

The lack of association between left atrial enlargement and OSA may be explained by the fact that obesity, hypertension, and diastolic dysfunction are all independently known to cause left atrial enlargement [15]. Also, studies done on association of left atrial enlargement with obstructive sleep apnea were done using more precise echocardiographic measurements of the left atrial diameter, rather than electrocardiographic features [15, 16].

Khalili et al. conducted a study in 1998 which included 190 patients and 50 controls and demonstrated 79% cases with terminal S wave in V5 and V6, 6% found to have right axis deviation, and 5% RBBB [11]. Our study similarly demonstrated a significant association of deep S waves in leads V5 and V6 with OSA patients. A possible explanation to this electrocardiographic feature is the hypertrophy of the crista supraventricularis leading to depolarizing forces directed superiorly and to the right. Crista supraventricularis is a ridge of tissue near the smooth walled portion of the right ventricular cavity that ends in the pulmonary trunk. The development of the hypertrophy of the crista supraventricularis seems to be a preamble to pulmonary hypertension [17, 18].

Unlike prior studies, our study found no association with QRS and QTc duration with OSA [8, 9]. An association with QTc duration would have provided an attractive explanation for increased arrhythmogenicity and incidence of cardiac sudden death.

Our study also tried to extrapolate the findings from a study that found a significant and direct association with atrial conduction time as defined by prolonged P wave duration [10]. This finding was postulated to signify a precursor of atrial fibrillation. We used the computer defined PR segment duration as a surrogate to the P wave duration, to determine if increasing severity of OSA had any significant association with P wave duration. This would help create an easy and rapid method to define patients diagnosed with OSA potentially at risk to develop atrial fibrillation. However, our study showed no significant association with P wave duration and OSA. The caveat of using the PR segment duration to define atrial conduction delay as opposed to the P wave duration is that the PR segment also includes the AV conduction delay period which may alter the association.

### 5.1. Limitations

The limitation to our study are: (1) Its retrospective design, which by its own nature does not provide the strongest correlations; however, most studies done prior were also retrospective. (2) Another limitation was the two demographic groups were not comparable to each other; however due to the nature of the study it would be difficult to compare all the demographic variables and also patients with OSA tend to have higher BMI and other comorbid conditions. (3) In our study, we could not establish an association between atrial fibrillation and OSA patients, which could possibly be because the majority of the patients have paroxysmal atrial fibrillation that might have been missed on a routine 10 second ECG. However, given the design of the study, this was one of the likely results.

## 6. Conclusion

This study could prove useful for primary prevention of cardiovascular diseases in general population. Our study could prove helpful not only for the sleep specialist, but also for primary care who could identify these ECG features in their office and use it as an auxiliary tool along with validated questionnaires in patients who are at high risk of OSA.

## Brief Summary

*Current Knowledge/Study Rationale*. Cardiac arrhythmias are commonly seen in patients with obstructive sleep apnea patient (OSA); however no definite electrocardiographic findings have been described in the literature that can be used for identifying patients with underlying OSA disease early in the course.

*Study Impact*. The aim of this study was to determine the relationship between specific electrocardiographic features and OSA. This may aid in early risk stratification of patients based on electrocardiographic features on a 12 lead ECG, a simple, cost-effective, and noninvasive test.

## Figures and Tables

**Table 1:** Comparison of demographic findings in patients diagnosed with obstructive sleep apnea and controls.

	**OSA (n = 215)**	**Control (n = 41)**	**p**
**Sex, Male, **%	93%	76%	<0.001

**Age, Median ± SD, years**	52.18 ± 14.04	44.55 ± 14.64	0.002

**BMI, median ± SD**	38.5 ± 9.9	36.5 ± 8.9	>0.005

**Obese (BMI >30), n (**%**)**	173 (80.4%)	29 (72.5%)	<0.05

**BMI <18.5, n (**%**)**	1 (0.40%)	0	>0.05

**BMI 18.5 – 24.9, n (**%**)**	7 (4.2%)	5 (12.5%)	>0.05

**BMI 25-29.9, n (**%**)**	34 (15.8%)	6 (15%)	>0.05

**BMI 30-34.9, n (**%**)**	27 (12.6%)	8 (20%)	<0.005

**BMI 35-39.9, n (**%**)**	52 (23.3%)	7 (17.5%)	<0.005

**BMI >40, n (**%**)**	94 (43.7%)	15 (37.5%)	<0.005

***Preexisting condition***			

**Chronic Obstructive**			>0.05
**Pulmonary Disease, n (**%**)**	12 (5.6%)	10%	

**Diabetes Mellitus, n (**%**)**	48 (22.3%)	20%	>0.05

**Hypertension, n (**%**)**	91 (42.3%)	37.5%	>0.05

***Sleep Study Findings***			>0.05

**AHI, median ±SD**	22 ± 30.4	1.4 ± 6.1	>0.05

**AHI 5-15, n (**%**)**	30.2%	0	>0.05

**AHI 16-30, n (**%**)**	23.2%	0	>0.05

**AHI >31, n (**%**)**	39.1	0	>0.05

**Table 2:** EKG findings in patients diagnosed with obstructive sleep apnea and controls.

	**OSA (n= 215)**	**Control (n= 40)**	**p**	**p, when age and gender** **controlled**
**Atrial Fibrillation, n (**%**)**	4 (1.9%)	0	0.413	

**Left Atrial**	33 (15.3%)	9 (22.5%)	0.157	
**enlargement, n (**%**)**				

**Right Ventricle**			>0.05	
**hypertrophy, n (**%**)**	0	1 (2.5%)		

**Left Ventricle**			>0.05	
**hypertrophy, n (**%**)**	32 (14.8%)	7 (17.5%)		

**Axis, Median ± SD**	15 ±37	27 ± 44.9	>0.05	

** Right axis deviation, **	5 (2.3%)	4(10%)	>0.05	
**(+90) - (+180), n (**%**)**				

** Left axis deviation, **	24 (11.2%)	0	>0.05	
** (-30) - (-90), n (**%**)**				

**Normal Axis, (+90) - (-90), n (**%**)**	186 (86.5%)	36 (90%)	>0.05	

**Deep S in V5-6, n (**%**)**	84 (39.1%)	8 (20%)	0.014	0.17

**RS pattern with Deep S in Leads I and AVF, n (**%**)**	95 (44.2%)	10 (25%)	0.017	0.14

**Atrial Premature**			>0.05	
**Contractions, n (**%**)**	6 (2.8%)	0		

**Left Anterior**			>0.05	
**Hemi block, n (**%**)**	13 (6%)	0		

**Premature Ventricular**			>0.05	
**Contractions, n (**%**)**	8 (3.7 %)	0		

**Atrioventricular Block, n (**%**)**	9 (4.2 %)	1 (2.5%)	>0.05	

**Right Bundle Branch**			>0.05	
**Block, n (**%**)**	8 (3.7 %)	0		

**Heart Rate, Median ±SD**	75 ±14.3	72 ± 19.5	>0.05	

**Bradycardia, n (**%**)**	23 (10.7 %)	6 (15%)	>0.05	

**Tachycardia, n (**%**)**	12 (5.6 %)	4 (10%)	>0.05	

**Normal Heart Rate, n (**%**)**	181 (84.2%)	30 (75%)	>0.05	

**QTc, Median ±SD**	434 ± 31.6	430 ± 79.8	>0.05	

**Prolonged QTc, n (**%**)**	8 (3.7 %)	5 (12.5%)	>0.05	

**PR, Median ±SD**	160 ± 28.3	158.5 ± 26.4	>0.05	

**QRS, Median ±SD**	92 ± 14.7	88 ± 8.9	>0.05	

## Data Availability

The DATA (tables) used to support the findings of this study are included in the article.

## Abbreviations

BMI: Body mass index
ECG: Electrocardiogram
IRB: Institutional Review Board
LVH: Left ventricular hypertrophy
OSA: Obstructive sleep apnea
PAC: Premature atrial contractions
PVC: Premature ventricular contractions
RVH: Right ventricular hypertrophy
RBBB: Right bundle branch block.

## Supplementary Materials

Supplementary Materials Electrocardiogram findings: (i) Left atrial enlargement was identified through electrocardiogram using standard definition as follows: if the length if the P wave in lead II is greater than 120 milliseconds, or if there is a downward deflection of the P wave in lead V1 is greater than 40 milliseconds in length, with greater than 1-millimeter negative deflection. (ii) Deep S wave. Usually the transition zone is in lead V3 and V4; however if S wave is more prominent and bigger than R wave then we define them as Deep S wave. (iii) LVH: We mostly used Sokolov-Lyon criteria (S wave depth in V1 + tallest R wave height in V5-V6 > 35 mm) and Modified Cornell criteria when the R wave in aVL is greater than 12mm in amplitude in males and 10 mm in females. (iv) RVH: We used standard definition for defining RVH that included the following: right axis deviation of +110° or more, dominant R wave in V1 (> 7mm tall or R/S ratio > 1), or dominant S wave in V5 or V6 (> 7mm deep or R/S ratio < 1). (v) RBBB: We used standard definition for defining RBB that included the following: broad QRS > 120 ms, RSR' pattern in V1-3 (‘M-shaped' QRS complex), or wide, slurred S wave in the lateral leads (I, aVL, V5-6).
