# Supplementary material for: Electrocardiographic Associations Seen with Obstructive Sleep Apnea
Source: Sleep Disord. 2019 Feb 27;2019:9704785. doi: 10.1155/2019/9704785 (PMC6415287; doi:10.1155/2019/9704785)
Supplement: Supplementary Materials — Electrocardiogram findings: (i) Left atrial enlargement was identified through electrocardiogram using standard definition as follows: if the length if the P wave in lead II is greater than 120 milliseconds, or if there is a downward deflection of the P wave in lead V1 is greater than 40 milliseconds in length, with greater than 1-millimeter negative deflection. (ii) Deep S wave. Usually the transition zone is in lead V3 and V4; however if S wave is more prominent and bigger than R wave then we define them as Deep S wave. (iii) LVH: We mostly used Sokolov-Lyon criteria (S wave depth in V1 + tallest R wave height in V5-V6 > 35 mm) and Modified Cornell criteria when the R wave in aVL is greater than 12mm in amplitude in males and 10 mm in females. (iv) RVH: We used standard definition for defining RVH that included the following: right axis deviation of +110° or more, dominant R wave in V1 (> 7mm tall or R/S ratio > 1), or dominant S wave in V5 or V6 (> 7mm deep or R/S ratio < 1). (v) RBBB: We used standard definition for defining RBB that included the following: broad QRS > 120 ms, RSR' pattern in V1-3 (‘M-shaped' QRS complex), or wide, slurred S wave in the lateral leads (I, aVL, V5-6). [file 9704785.f1.pdf]

Electrocardiogram findings:

- Left atrial enlargement were identified through electrocardiogram using standard definition as: if the length if the P wave in lead II is greater than 120 milliseconds, or if there is a downward deflection of the P wave in lead V1 is greater than 40 milliseconds in length, with greater than 1-millimeter negative deflection.

- Deep S wave. Usually the transition zone is in lead V3 and V4, however if S wave are more prominent and bigger than R wave then we define them as Deep S wave.

- LVH: We mostly used Sokolov-Lyon criteria (S wave depth in V1 + tallest R wave height in V5-V6 > 35 mm), and Modified Cornell criteria when the R wave in aVL is greater than 12mm in amplitude in males and 10 mm in females.

- RVH: We used standard definition for defining RVH that included: Right axis deviation of +110° or more, dominant R wave in V1 (> 7mm tall or R/S ratio > 1), or dominant S wave in V5 or V6 (> 7mm deep or R/S ratio < 1).

- RBBB: We used standard definition for defining RBB that included: Broad QRS > 120 ms, RSR’ pattern in V1-3 (‘M-shaped’ QRS complex), or wide, slurred S wave in the lateral leads (I, aVL, V5-6)
